# The Fecal Microbiota in the Domestic Cat (*Felis catus*) Is Influenced by Interactions Between Age and Diet; A Five Year Longitudinal Study

**DOI:** 10.3389/fmicb.2018.01231

**Published:** 2018-06-19

**Authors:** Emma N. Bermingham, Wayne Young, Christina F. Butowski, Christina D. Moon, Paul H. Maclean, Douglas Rosendale, Nicholas J. Cave, David G. Thomas

**Affiliations:** ^1^Food Nutrition and Health Team, AgResearch, Palmerston North, New Zealand; ^2^High-Value Nutrition National Science Challenge, Auckland, New Zealand; ^3^Riddet Institute, Massey University, Palmerston North, New Zealand; ^4^School of Agriculture and Environment, Massey University, Palmerston North, New Zealand; ^5^Rumen Microbiology Team, AgResearch, Palmerston North, New Zealand; ^6^Bioinformatics and Statistics Team, AgResearch, Lincoln, New Zealand; ^7^Food Nutrition and Health Group, The New Zealand Institute for Plant and Food Research Ltd, Food Industry Science Centre, Palmerston North, New Zealand; ^8^School of Veterinary Science, Massey University, Palmerston North, New Zealand

**Keywords:** dietary format, canned diet, kibbled diet, insulin sensitivity index, aging, carnivore, body composition

## Abstract

In humans, aging is associated with changes in the gastrointestinal microbiota; these changes may contribute to the age-related increase in incidence of many chronic diseases, including Type 2 diabetes. The life expectancies of cats are increasing, and they are also exhibiting the same types of diseases. While there are some studies investigating the impacts of diets on gastrointestinal microbiota in young cats, the impacts of aging in older cats has not been explored. We followed a cohort of related kittens, maintained on two commercial diets (kibbled and canned) from weaning (8 weeks) to 5 years of age (260 weeks). We hypothesized that the long-term feeding of specific diet formats would (a) lead to microbial composition changes due to aging, (b) impact body composition, and (c) affect insulin sensitivity in the aging cat. We observed that both diet and age affected fecal microbial composition, and while age correlated with changes in body composition, diet had no effect on body composition. Similarly insulin sensitivity was not affected by age nor diet. 16S rRNA sequencing found unclassified *Peptostreptococcaceae* were prominent across all ages averaging 21.3% of gene sequence reads and were higher in cats fed canned diets (average of 25.7% of gene sequence reads, vs. 17.0% for kibble-fed cats). Age-related effects on body composition and insulin sensitivity may become apparent as the cats grow older; this study will continue to assess these parameters.

## Introduction

Companion animals are starting to mirror humans in terms of longer lifespans and acquisition of age-related health problems. Aging in cats is typically defined as: Kitten: < 6 months of age. Young adult: 7 months - 2 years of age Adult: 3–6 years of age. Mature: 7–10 years of age. Senior: 11–14 years of age. Geriatric: >15 years of age (Hoyumpa Vogt et al., 2010). It is currently estimated that 20% of pet cats are now older than 11 years of age (Bellows et al., 2016). As with humans, pet cats are facing increased health problems including an increased incidence of obesity and related illnesses (e.g., diabetes) (Scarlett et al., 1994; German, 2006; Banfield Pet Hospital, 2016). The intestine may play a central role in these conditions; it is the site of nutrient absorption, and is a barrier between the host and external pathogens and toxins. Intestinal bacteria play a pivotal role in maintaining health and well-being of their host, whether human or pet. Indeed, in humans, dysbiosis is linked to diseases such as obesity (Turnbaugh et al., 2006) and Type 2 diabetes (Larsen et al., 2010; Zhang et al., 2013). Therefore, healthy intestinal function, including the activity of the resident microbiota, is likely to influence the health, wellbeing and longevity of companion animals.

Aging influences intestinal function in many species. In the human intestinal tract, aging is associated with changes in the microbiota beginning with initial colonization and development toward maturation in early life, relative stability in adult life, and decreased diversity in elderly life (Kumar et al., 2016). Age-related microbiome changes may contribute to the increase in intestinal-related conditions such as slower transit time in both humans and cats, and decreased nutrient digestibility in older cats (Peachey et al., 1999; Bermingham et al., 2012). While there are a number of studies that have investigated the impacts of age on gastrointestinal microbiota in kittens and young-adult cats (< 2 years of age; Jia et al., 2011b; Bermingham et al., 2013a; Hooda et al., 2013; Deusch et al., 2015), mature and senior (8–14 years of age; Jia et al., 2011a), these studies are often confounded by differences in diet, animal to animal variation and the methodologies used to analyse the microbiome. To our knowledge, there are no published longitudinal studies of how the microbiome changes in animal cohorts beyond 1 year of age.

The incidence of Type 2 diabetes is rare, but increasing in the domestic cat (Ohlund et al., 2015; Banfield Pet Hospital, 2016). Type 2 diabetes is typically characterized by a decrease in the efficacy of insulin in the body; i.e., reduced insulin sensitivity. Reduced insulin sensitivity is a precursor for the development of Type 2 diabetes in the cat (Feldhahn et al., 1999). The impact of diet, specifically its carbohydrate content, and its potential contribution to developing Type 2 diabetes in the cat, has long been of interest, and has recently been extensively reviewed (Verbrugghe and Hesta, 2017). Pet cats are typically fed kibbled or canned diets which differ in macronutrient profiles: kibbled diets have lower protein and higher carbohydrate levels, whereas canned diets have very low carbohydrate content, and medium to high levels of protein and fat (Davies et al., 2017). While high levels of both protein and carbohydrate intake alter insulin sensitivity (Verbrugghe et al., 2010), recent epidemiological evidence suggests that kibbled diets are a risk factor for diabetes in non-obese cats (Öhlund et al., 2017). While the risk associated with Type 2 diabetes varies with breed, cats typically have increased risk of developing Type 2 diabetes from around 6 years of age, with the mean age of diagnosis being 10 years old (Ohlund et al., 2015; O'Neill et al., 2016). The risk of reduced insulin sensitivity is thought to increase with age (Hoenig et al., 2011) and body condition score (Häring et al., 2013). To our knowledge, only one study has looked at the effects of age on insulin sensitivity in the domestic cat: Backus and colleagues observed that while insulin sensitivity tended to decrease in cats older than 4 years (from 4 to 7 years of age) compared to a separate group of younger cats (range: 0.8–2.3 years of age), this effect was not observed when adjusted for body weight (Backus et al., 2010). While this suggests aging *per se* does not increase risk of developing Type 2 diabetes, long-term, controlled studies on the cat are lacking.

As with humans, the gastrointestinal microbiome of cats plays an important part in their overall health and disease. We know that diet and disease both impact the microbiome of cats. Typically, we use a range of intestinal-related biomarkers of health including short-chain fatty acid concentrations, fecal microbiota composition and predictive function of the fecal microbiome to assess the impacts of diets on pet health, which are typically derived from human and/or rodent studies. However, carnivorous pets have a very different physiology to omnivorous humans and rodents. Despite this, currently the majority of data used to advance the understanding of the relevance of specific microbial groups is from a human (omnivore) microbiome perspective, with microbiome functions characterized and modeled from this. Recently, our and others' research has shown that dogs do not always respond to dietary ingredients (Bermingham et al., 2017) and intestinal disease (Vázquez-Baeza et al., 2016) the same as humans. This raises the question of how applicable microbiome interpretations from an omnivore's or herbivore's perspective can be directly applied in dogs and cats.

We aimed to determine the impacts of both diet and aging on fecal microbial composition, body composition and insulin sensitivity on a single cohort of cats. We followed a cohort of related (siblings) kittens, maintained on two commercial diets (kibbled and canned) from weaning (8 weeks) to 5 years of age (260 weeks). We hypothesized that the long-term feeding of specific diet formats would (a) lead to microbial composition changes due to aging, (b) impact body composition, and (c) affect insulin sensitivity in the aging cat. To better understand how diet and the microbiome interact in the cat, we undertook a dataset integration approach using the 260 week data. Data pertaining to fecal microbial composition at 8 and 17 weeks have been previously published (Bermingham et al., 2013a; Young et al., 2016).

## Materials and methods

### Ethics statement

All procedures undertaken on the cats were performed in accordance with relevant guidelines and regulations of the Animal Welfare (Companion Cats) Code of Welfare (2007) and approved by the Massey University Animal Ethics Committee (8, 17, 62 weeks—MUAEC 10/108 and 260 weeks—MUAEC 16/28). All cats used were owned by Massey University and were housed at the Centre for Feline Nutrition (Massey University, Palmerston North, New Zealand).

### Animals and diets

Animals, weaning and housing have been described in detail previously (Bermingham et al., 2013a). Briefly, eight queens (mean age 5.3 ± 0.4 year) were randomly allocated either a kibbled or canned (*n* = 4 per diet) diet. Each queen was mated with a single unrelated male fed with the same diet. Half of each litter was randomly assigned (within sex) onto the kibbled diet (*n* = 6 females and 2 males) and half onto a canned diet (*n* = 7 females and 3 males). The cats were colony-housed according to diet at 8 weeks of age and remained in these groups for the duration of the study. All cats used in the study were neutered at 36 weeks (approximately 9 months) of age.

Commercially available kibbled (moderate protein:fat:carbohydrate−35:20:28% dry matter (DM); *n* = 9 cats) and canned (protein:fat:carbohydrate−45:37:2%DM; *n* = 9 cats) diets were utilized in this study (Table 1). Both diets were formulated to meet the nutrient requirements for growth, gestation and lactation according to the Association of American Feed Control Officials (2016). The diets were commercially manufactured to meet stated (on pack) macronutrient levels. Therefore, there will be batch to batch variations that we have not been able to account for. However, it is probable that batch to batch variations in macronutrient profiles are less than the differences between kibbled and canned diets. Diet and water were available *ad libitum* daily for the duration of the study.

**Table 1:** Macronutrient profiles of test diets used at 17 and 260 weeks.

	**17 weeks**		**260 weeks**	
	**Kibbled**	**Canned**	**Kibbled**	**Canned**
Dry matter (%)	92.9	31.7	94.7	20.8
Crude Protein (%)	35.3	45.3	39.8	51.5
Crude Fat (%)	20.2	37.6	19.2	33.9
Crude Fiber (%)	1.8	1.5	1.2	1.2
Ash (%)	7.5	7.2	7.8	8.2
NFE (%)	28.2	2.0	32.0	5.2
Gross energy (kJ/g)	21.2	25.9	21.3	24.7

*Both diets were commercially available, Association of American Feed Control Officials (AAFCO) formulated diets. NFE, Nitrogen free extractables, calculated by difference: 100%—crude protein %—crude fat %—crude fiber %—ash %)*.

### Digestibility

At 17 and 260 weeks of age, the apparent digestibility of dietary macronutrients (gross energy, crude protein and crude fat) was determined. Individual total food intake and refusals were recorded daily and total fecal output was collected over a 5 day period (Association of American Feed Control Officials, 2016). Sub-samples of diets and total feces collected were frozen (−20°C), freeze dried, and finely ground for analysis. The diet and feces were analyzed for moisture content by drying to constant weight using a convection oven at 105°C (AOAC 930.15, 925.10). Ash weight was measured after heating in a furnace at 550°C (AOAC 942.05). Crude protein and crude fat levels were determined using the Leco total combustion method (AOAC 968.06) and acid hydrolysis/Mojonnier extraction (AOAC 954.02), respectively. Gross energy was determined using bomb calorimetry. Crude fiber was determined using the gravimetric method (AOAC 978.10) and Nitrogen Free Extractables (NFE) was calculated by difference (Bermingham et al., 2012).

### Fecal sampling

At 8, 17, 104, and 260 weeks of age, the kittens were housed individually for 24 h and a fresh fecal sample was collected from each animal within 15 min of excretion, snap-frozen in liquid nitrogen and stored at −80°C for subsequent analysis.

#### Fecal organic acids

Fecal samples were diluted 1:5 with phosphate-buffered saline containing 2-ethylbutyric acid as an internal standard. Fecal aqueous extracts were analyzed as described previously (Richardson et al., 1989). Briefly, aqueous extracts were acidified, phase separated into diethyl ether and stored at −80°C until analysis. Organic acids were derivatised with N-tert-butyldimethylsilyl-N-methyltrifluoroacetamide plus 1% tert-butyldimethylchlorosilane (99:1; Sigma-Aldrich) and analyzed on a Shimadzu capillary gas chromatography (GC) system (GC-2010 Plus, Tokyo, Japan) equipped with a flame ionization detector (FID) and fitted with a Restek column (SH-Rtx-1, 30 m × 0.25 mm ID × 0.25 μm) using helium as the carrier gas. The GC-FID was controlled and data processed using Shimadzu GC Work Station LabSolutions Version 5.3, with sample organic acids quantified in reference to authentic standards.

#### Fecal microbial composition

DNA was extracted from fecal samples using the NucleoSpin Soil kit (Macherey Nagel, Düren, Germany) following the manufacturer's instructions. Fecal microbiota profiles were determined by Illumina MiSeq sequencing of the V4–V6 region of the bacterial 16S rRNA gene, as described previously (Young et al., 2015).

Fecal microbial amplicon sequences were processed using Qiime 1.8 (Caporaso et al., 2010). Reads were quality filtered using default settings and sequences were chimera-checked using the USEARCH method against aligned sequences from the Greengenes database (release GG_13_8). Chimeric sequences were removed from subsequent analyses. Sequences were clustered at 97% similarity into operational taxonomic units (OTUs) using the UCLUST method and representative sequences were assigned taxonomies using the Ribosomal Database Project (RDP) classifier. Differences between mean relative abundance of taxa between treatments were analyzed by two-factor ANOVA (diet × time) and single factor (diet) permutation ANOVA using the RVAideMemoire R package with 2,000 permutations (Hervé, 2014). R codes for the microbial analysis are provided in Supplementary Material, Data Sheet 1. The mean number of paired-end reads after quality filtering was 37,344, with a minimum of 16,053, maximum of 103,888, and a standard deviation of 15,338. Sequences were submitted to NCBI Sequence Read Archive (reference PRJNA470724).

### Body composition

At 62 and 260 weeks of age, food was withheld from the cats 12–18 h before sedation via intravenous infusion of ketamine hydrochloride (5 mg/kg) and diazepam (0.25 mg/kg). A single port central venous jugular catheter was placed using sterile technique and a baseline jugular venous blood sample (0.5 ml) collected. A single cutaneous stay suture was placed and the site was dressed with iodine and sterile gauze. Cats were then given *ad libitum* access to food through the day before an overnight fast (12–18 h) ready for sampling. This catheter was used to determine body composition, and undertake an intravenous glucose tolerance test (IVGTT) and intravenous insulin tolerance test (IVITT).

The following day, blood samples were collected (2 ml) immediately prior (T0) to deuterium oxide (0.4 g/kg bodyweight made isotonic with saline) being injected into a 20 gauge cephalic venous catheter (Backus et al., 2000). A blood sample was collected 2 h after injection and plasma was separated and frozen at −20°C until analysis. Water was available at all times, except for during the 2 h of equilibrium after D_2_O injection.

#### Plasma enrichment of ^2^H

In order to determine body composition, the enrichment of ^2^H in water was determined by transfer of the hydrogen in water to acetylene and subsequent analysis of the acetylene isotopes by isotope-ratio mass spectrometry (IRMS) (Van Kreel et al., 1996). This method was adapted and modified by converting the acetylene eluting from the gas chromatograph to hydrogen and analyzing the resulting hydrogen. Briefly, 350 mg of granulated calcium carbide (Sigma) was transferred into a 12 ml executainer (Labco, UK), sealed with a cap and septa, and evacuated. Plasma (20 μl) was injected through the septa onto the bed of calcium carbide and allowed to react at room temperature for a minimum of 30 min before analysis on a GC-Thermal Chromatography-IRMS. The determination of ^2^H enrichment for the headspace acetylene derived from plasma was carried out with a Thermo Finnigan Delta V Plus continuous flow isotope ratio mass spectrometer (Thermo-Finnigan, Bremen, Germany) coupled online with a Thermo Trace GC via a Thermo Conflow III combustion interface (a high temperature pyrolysis furnace at 1,450°C). Acetylene eluting from the GC column was pyrolysed to H_2_. After drying the gas stream by a nafion membrane, gases were introduced into the IRMS ion source. A capillary column (Agilent Poraplot Q, 30 m × 0.32 mm ID) with helium as carrier gas (1.5 ml/min) was used for the separation of acetylene from air components. Fifteen microliters was injected in the split mode (1:20 spilt ratio) by an autosampler (CTC A200S; CTC analytics, Zwingen, Switzerland) fitted with a 100 μl headspace syringe. The column head pressure was 150 kPa and injector temperature 110°C. The GC oven temperature was maintained isothermally at 110°C for the duration of the run. Data processing was performed by the vendor provided software, ISODAT. For purposes of IRMS calibration, mixtures of D_2_O and unlabelled H_2_O, between 0 and 5 mg of D_2_O per ml H_2_O, were prepared and analyzed in analogous fashion to the plasma samples. Body composition was calculated as described previously, using total body mass divided by 0.744 to account for water content of lean mass in cats (Backus et al., 2000).

#### IVGTT and IVITT

Immediately after the initial D_2_O injection, 1 g/kg bodyweight of glucose solution (50 g/dl) was infused into the cephalic venous catheter over 60 s (Backus et al., 2010). Blood samples were collected at 5, 10, 15, 30, 45, 60, 90, and 120 min after from the central venous jugular catheter. Cats were then offered *ad libitum* access to food through the day before an overnight fast (14–18 h). The next day, 0.01 U/kg recombinant human insulin (Actrapid®) was injected into the cephalic venous catheter. Immediately before injection, and 5, 10, 15, and 20 min after, blood samples (1 ml) were taken from the central venous jugular catheter. The cats were offered *ad libitum* access to food post-sampling.

The blood samples were placed on ice until plasma was harvested (10 min at 4°C, at 1,200 *g*). Plasma levels of glucose (hexokinase) and insulin (radioimmunoassay) were measured. Area under the curve (AUC) and insulin sensitivity indices were assessed as described previously (Backus et al., 2010).

### Statistical analysis

Body weight, lean body mass, fat mass, apparent nutrient digestibility, fecal weight and glycaemic responses were analyzed using a 2-way (diet and week) repeated measures Correlation Models by REML using diet (canned vs. kibbled) and time (age) as factors (Genstat version 17.1). The results from the insulin assay were unreliable and are not reported in this paper. For the glucose parameters, due to catheter failure, two data points are missing for the canned treatment group at 104 weeks; additionally for the 260 week data one data point is missing for each the canned and kibbled diet.

Only data relating to the cats at 260 weeks of age were integrated using similar methodology described previously (Bermingham et al., 2017). Briefly, physiological measurements (body fat %, body lean %, insulin sensitivity index, apparent macronutrient digestibility, and fecal organic acids) and microbial operational taxonomic units (OTU) frequencies were compiled. This resulted in a dataset with 16 samples matched by cat and time point. To ensure that there was sufficient variability for the analysis and integration of the dataset, the “nearZeroVar” function in the mixOmics version 6.3.0 R package (González et al., 2012) was applied. Following the filtering of low-variance taxa and physical measurements, principal component analyses (PCAs) were performed using standard the R command (Team, 2015). Two-factor (pre-diet × post-diet) permutation ANOVA was then completed on each remaining variate for each dataset using the lmPerm version 2.1.0 R package. The mixOmics R package was used to perform shrunk canonical correlation analysis (CCA) for the integration of fecal 16S rDNA amplicon data and combined metabolomic and physical measurement data to account for a large amount of co-correlation between the metabolomic and physical measurement data. The resulting association measures between pairs of variates were defined as: >|0.8| high, >|0.6| good, and >|0.50| moderate associations, and can be interpreted in a similar manner to a correlation *R*^2^-value. In order to better understand the relationship between insulin sensitivity index and fecal microbiota insulin sensitivity values were overlaid with microbiota generating Unifrac PCoA plots. For this, only cats where we had data for at least 3 of the time points (8, 17, 62, and 260 weeks) were analyzed.

## Results

### Bodyweight and lean body mass

Bodyweight, lean body mass and fat mass are shown in Table 2. There was no significant effect of diet on bodyweight, lean mass, or fat mass in the cats (Table 2). Age had a significant effect (*P* < 0.001) on bodyweight and lean mass with older cats (260 weeks) being heavier and leaner than when they were 62 weeks of age. There was no significant effect of age on fat mass (Table 2).

**Table 2:** Bodyweight (kg), lean mass (kg), fat mass (kg), and insulin sensitivity index* in cats (*Felis catus*) fed either kibbled (*n* = 9) or canned (*n* = 8) diets at 62 and 260 weeks of age.

	**Kibbled**				**Canned**						
	**62 weeks of age**		**260 weeks of age**		**62 weeks of age**		**260 weeks of age**		***P*****-value**		
	**Mean**	**SEM**	**Mean**	**SEM**	**Mean**	**SEM**	**Mean**	**SEM**	**Diet**	**Week**	**Diet × week**
Bodyweight (kg)	3.4	0.2	4.0	0.3	3.3	0.2	4.0	0.3	0.783	< 0.001	0.632
Lean Mass (kg)	2.6	0.2	3.2	0.2	2.7	0.1	3.2	0.2	0.996	< 0.001	0.620
Fat Mass (kg)	0.8	0.1	0.8	0.2	0.6	0.1	0.8	0.1	0.538	0.138	0.256
% Lean	78	1.8	82	2.0	82.7	1.7	81.6	2.2	0.344	0.324	0.106
% Fat	22	1.8	18	2.0	17.3	1.7	18.4	2.2	0.344	0.324	0.106
Glucose T0 (mmol/l)	5.3	0.3	5.4	0.3	5.1	0.1	6.1	0.2	0.463	0.003	0.032
Glucose T15 (mmol/l)	4.2	0.4	4.4	0.4	4.5	0.3	4.9	0.3	0.516	0.072	0.824
Insulin Sensitivity Index	0.2	0.1	0.2	0	0.1	0.1	0.2	0	0.750	0.686	0.473

Results are reported as mean and associated standard error of the mean (SEM).

* For the glucose and insulin parameters two data points are missing for the canned treatment group at 62 weeks; additionally for the 260 week data one data point is missing for each the canned and kibbled diet.

† Insulin sensitivity index = [Glucose]_T0_-[Glucose]_T15_/[Glucose]_T0_.

*SEM, standard error of the mean*.

### Glucose and insulin tolerance tests

**Figure 1:**
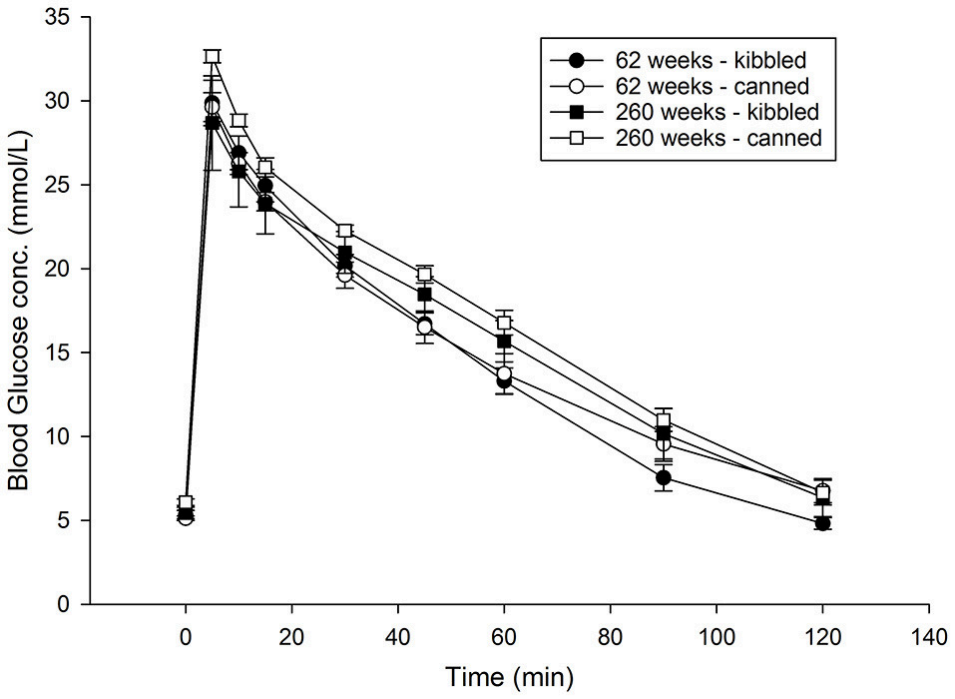
Blood glucose concentrations following an intravenous glucose tolerance test (IVGTT) in domestic cats (*Felis catus*) fed either kibbled (*n* = 8) or canned (*n* = 8) diets at 62 and 260 weeks of age. Results are reported as means and error bars represent the standard error of the mean.

Plasma glucose concentrations from the IVGTT performed on 62 and 260 weeks old cats are shown (Figure 1). There was no effect of diet on either glucose concentrations or AUC (data not shown). While the concentration of plasma glucose was lower in the younger cats at 30 (*P* = 0.08), 45 (*P* = 0.02), 60 (*P* = 0.02), and 90 min (*P* = 0.08), there were no significant effects due to age on the total AUC for glucose.

**Figure 2:**
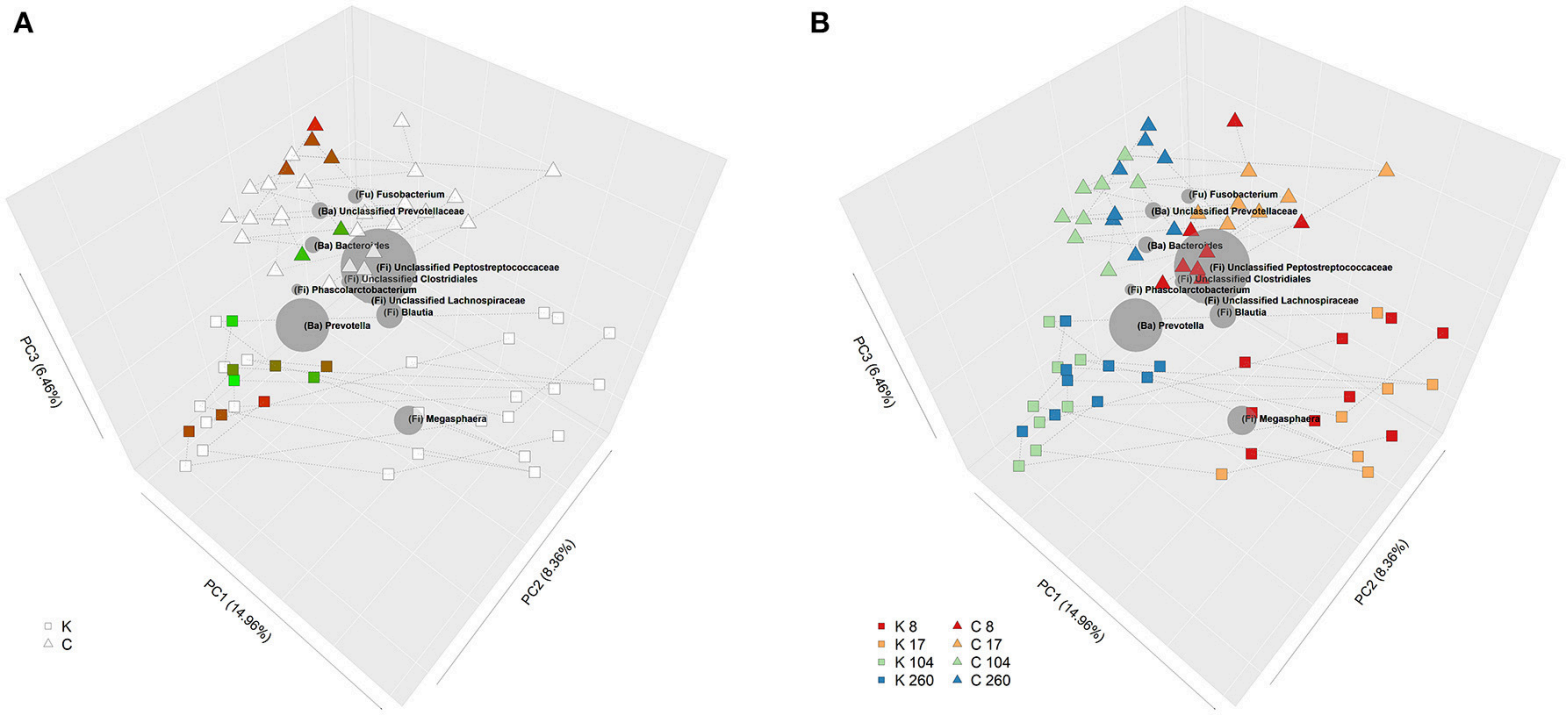
**(A)** Bi-plot showing principle coordinate analysis (PCoA) of unweighted Unifrac phylogenetic distances of fecal communities in domestic cats (*Felis catus*) fed either canned or kibbled diets at 8, 17, 104, and 260 weeks of age. Shape indicates diet; square (kibbled; K), triangle (canned; C). Color of squares and triangles represent insulin sensitivity with green representing low insulin sensitivity and red high insulin sensitivity. Lines join samples from the same cat. **(B)** Bi-plot showing PCoA of unweighted Unifrac phylogenetic distances of fecal communities in domestic cats (*Felis catus*) fed either canned or kibbled diets at 8, 17, 104, and 260 weeks of age. Shape indicates diet; square (kibbled; K), triangle (canned; C). Color of squares and triangles indicate age; red (week 8), orange (week 17), green (week 104), blue (week 260). Lines join samples from the same cat. Size of gray spheres, representing taxa, are proportional to the average relative abundance across all samples. Position of gray spheres relative to samples indicate their degree of correlation between taxa and samples; i.e., the closer the taxon is to particular samples, the higher the relative abundance of that taxon in those samples.

Insulin sensitivity index, calculated from the IVITT was unaffected by diet or age (Table 2). Insulin sensitivity data were overlaid on the Unifrac PCoA plots, which showed some delineation between dietary groups; in general, insulin sensitivity index appeared to be higher in kibble-fed cats (Figure 2A).

### Apparent nutrient digestibility

The apparent nutrient digestibility parameters for the cats at 17 weeks of age have been reported previously (Young et al., 2016). Regardless of age, diet affected the apparent digestibility of crude protein and dry matter with these being higher in cats fed canned diets (Table 3). The apparent digestibility of crude fat, crude protein, and gross energy increased as the cats aged (Table 3). There was a significant (*P* < 0.05) interaction between diet and age for the apparent digestibility of crude protein, gross energy, and dry matter (Table 3). Fecal dry matter was lower in cats fed the canned diets (*P* < 0.05), however it was unaffected by age of the cat (Table 3).

**Table 3:** Apparent digestibility of protein, fat, energy, and dry matter (DM) and fecal concentrations of organic acids in domestic cats (*Felis catus*) fed either kibbled (*n* = 9) or canned (*n* = 9) diets at 17, 104, and 260 weeks of age.

		**Kibbled**						**Canned**								
		**17 weeks of age**		**104 weeks of age**		**260 weeks of age**		**17 weeks of age**		**104 weeks of age**		**260 weeks of age**		***P*****-value**	
		**Mean**	**SEM**	**Mean**	**SEM**	**Mean**	**SEM**	**Mean**	**SEM**	**Mean**	**SEM**	**Mean**	**SEM**	**Diet**	**Week**	**Diet × week**
Digestibility Coefficient																
	Fat	0.819	0.017			0.931	0.015	0.862	0.026			0.935	0.007	0.15	< 0.001	0.34
	Protein	0.723	0.015			0.852	0.011	0.879	0.007			0.876	0.007	< 0.001	< 0.001	< 0.001
	Energy	0.735	0.015			0.786	0.014	0.822	0.014			0.807	0.009	0.08	0.003	0.01
	Dry matter	0.745	0.014			0.840	0.012	0.810	0.011			0.817	0.008	< 0.001	0.171	0.02
Fecal dry matter		33	1.4	33.1	1	34.1	2.9	26.2	1.4	28.8	1.3	31.4	2.6	0.03	0.39	0.73
Organic Acid (μmol/g DM)																
	Acetate	188	56	192	16.1	312	36.4	400	56	209	44.9	186	30.9	0.48	0.497	0.72
	Butyrate	158	19	68.8	6.0	57.1	5.9	98	19	97.1	8.5	84.2	10.8	0.16	< 0.001	< 0.001
	Propionate	89	19.3	106	8.6	143	15.8	149	19.3	127	15.9	95.4	12.6	0.97	0.992	0.01
	Formate	0.9	0.05	0.91	0.03	0.92	0.06	1.2	0.05	1.06	0.05	1.11	0.15	0.02	0.927	0.89
	Lactate	0.9	0.05	0.9	0	1.2	0.2	1.2	0.05	1.1	0	1.6	0.2	0.09	0.031	0.80
	Valerate	174	28.2	68.8	8.4	30.7	5.7	43	28.2	75.9	11.1	45.7	7.2	0.15	< 0.001	< 0.001
	Isobutyrate	16.3	2.9	13.8	1.4	6.0	0.7	24.1	2.9	22	2.9	17.6	2.0	< 0.001	0.001	0.65
	Isovalerate	45.4	8.7	27.6	3.2	10.4	1.4	46.7	8.7	43.4	5.3	35.1	3.9	< 0.001	< 0.001	0.11
	Hexanoate	19.8	4.1	2.9	0.8	0.6	0.2	0.4	4.1	2.5	2.2	0.3	0	0.01	< 0.001	< 0.001
	Heptanoate	0.3	0.02	0.3	0	0.3	0	0.4	0.02	0.4	0	0.3	0	0.01	0.71	0.63
	Succinate	3.1	0.97	0.9	0	0.9	0.1	1.2	0.97	1.1	0	1	0.1	0.29	0.003	0.01

*Results are reported as mean and associated standard error of the mean (SEM). DM, dry matter*.

### Fecal organic acids

The concentrations of fecal organic acids are presented in Table 3. On average, cats fed the canned diet had higher fecal concentrations of formate, isobutyrate, isovalerate, and heptanoate than cats fed the kibbled diets (*P* < 0.05). In contrast, hexanoate was decreased (*P* < 0.05) in cats fed the canned diets. Age affected the concentration of a number of organic acids: butyrate, valerate, isobutyrate, isovalerate, hexanoate, and succinate all decreased with age whereas lactate concentrations increased with age (*P* < 0.05). The concentrations of butyrate, propionate, valerate, hexanoate, and succinate all showed significant diet and age interactions (Table 3).

### Fecal microbial composition

The fecal microbiota composition of kittens at weeks 8 and 17, determined by 454 amplicon sequencing and shotgun metagenome sequencing, have been reported previously (Bermingham et al., 2013a; Young et al., 2016). To directly compare data from these time points with those at the latter time points, the DNA samples from the week 8 and 17 samplings were re-analyzed using the same Illumina MiSeq V3–V4 16S amplicon sequencing as that used for week 102 and 260 samples.

Community profiles clearly clustered according to diet and age (Figure 2B). Of the 50 most abundant taxa at the genus level or higher (Table 4), which comprised 98.9% of sequence reads, 33 were significantly affected by diet [false discovery rate (FDR) < 0.05], 27 were significantly affected by age (FDR < 0.05), and 18 had a significant age by diet interaction (FDR < 0.05).

**Table 4:** Abundance of genus-level taxa (percent of total sequences) in the fecal microbiota of domestic cats (*Felis catus*) fed either kibbled (*n* = 9) or canned (*n* = 9) diet at 8, 17, 104, and 260 weeks of age.

			**Kibbled**								**Canned**													
			**8 weeks of age**		**17 weeks of age**		**104 weeks of age**		**260 weeks of age**		**8 weeks of age**		**17 weeks of age**		**104 weeks of age**		**260 weeks of age**		***P*****-value**			**FDR**		
**Phylum**	**Family**	**Genus**	**Mean**	**SEM**	**Mean**	**SEM**	**Mean**	**SEM**	**Mean**	**SEM**	**Mean**	**SEM**	**Mean**	**SEM**	**Mean**	**SEM**	**Mean**	**SEM**	**Diet**	**Week**	**Diet × Week**	**Diet**	**Week**	**Diet × Week**
***Actinobacteria***																								
	* Bifidobacteriaceae*	*Bifidobacterium*	2.950	0.676	4.522	1.351	0.166	0.135	0.175	0.082	0.001	0.000	0.001	0.001	0.000	0.000	0.000	0.000	0.000	0.000	0.000	0.000	0.000	0.000
	* Coriobacteriaceae*	*Asaccharobacter*	0.078	0.026	0.184	0.045	0.284	0.047	0.144	0.027	0.084	0.034	0.024	0.012	0.015	0.003	0.091	0.017	0.000	0.135	0.000	0.000	0.190	0.000
		*Collinsella*	0.727	0.159	1.144	0.156	0.202	0.060	0.297	0.103	0.092	0.035	0.066	0.021	0.017	0.006	0.042	0.009	0.000	0.000	0.002	0.000	0.000	0.008
***Bacteroidetes***																								
	* Bacteroidaceae*	*Bacteroides*	2.963	1.170	0.078	0.033	3.170	0.978	1.107	0.219	9.664	1.479	1.841	0.712	12.505	3.229	5.367	1.887	0.000	0.000	0.119	0.000	0.000	0.248
	Uncl. *Bacteroidales*	Uncl. *Bacteroidales*	0.015	0.007	0.001	0.001	0.059	0.018	0.022	0.005	0.122	0.029	0.119	0.034	0.191	0.022	0.098	0.020	0.000	0.002	0.555	0.000	0.005	0.650
	* Porphyromonadaceae*	Uncl. *Porphyromonadaceae*	0.062	0.039	0.010	0.010	0.089	0.025	0.042	0.012	0.222	0.085	0.050	0.018	0.063	0.014	0.175	0.050	0.006	0.039	0.177	0.011	0.067	0.328
		*Parabacteroides*	0.284	0.157	0.004	0.004	0.139	0.039	0.011	0.003	0.173	0.051	0.040	0.024	0.038	0.011	0.038	0.018	0.458	0.002	0.723	0.573	0.005	0.785
	* Prevotellaceae*	Uncl. *Prevotellaceae*	0.769	0.308	0.150	0.087	2.126	0.839	1.858	0.384	7.932	0.991	6.096	1.413	7.948	1.734	10.274	1.450	0.000	0.047	0.555	0.000	0.076	0.650
		*Prevotella*	12.856	2.192	7.791	3.147	21.344	2.878	38.136	4.368	11.917	2.347	6.815	1.632	8.532	1.903	16.021	3.390	0.000	0.000	0.001	0.000	0.000	0.003
***Firmicutes***																								
	* Enterococcaceae*	*Enterococcus*	2.433	2.086	2.772	1.793	0.010	0.010	0.006	0.005	0.350	0.296	0.283	0.165	0.005	0.002	0.081	0.055	0.533	0.167	0.584	0.629	0.220	0.663
	* Lactobacillaceae*	*Lactobacillus*	6.849	1.828	10.826	2.364	0.000	0.000	0.043	0.025	0.002	0.002	0.002	0.001	0.000	0.000	0.000	0.000	0.000	0.000	0.000	0.000	0.000	0.000
	* Streptococcaceae*	*Lactococcus*	1.188	0.508	0.000	0.000	0.000	0.000	0.007	0.007	0.014	0.006	0.021	0.014	0.000	0.000	0.000	0.000	0.010	0.000	0.000	0.017	0.000	0.000
		*Streptococcus*	5.164	3.222	2.686	2.563	1.117	1.109	0.016	0.013	0.004	0.002	0.071	0.062	0.001	0.000	0.001	0.001	0.026	0.387	0.249	0.040	0.440	0.402
	* Clostridiaceae*	*Clostridium*	0.737	0.616	0.103	0.063	0.187	0.107	0.802	0.393	4.167	1.796	10.033	4.073	1.994	0.655	3.159	1.200	0.000	0.049	0.029	0.000	0.077	0.075
	* Eubacteriaceae*	*Eubacterium*	0.499	0.320	0.827	0.112	1.234	0.359	0.432	0.133	1.392	0.318	2.094	0.426	2.516	0.248	2.874	0.355	0.000	0.014	0.053	0.000	0.026	0.121
	* Clostridiales Incertae Sedis XIV*	*Blautia*	10.263	4.093	13.056	2.895	6.465	0.824	8.708	0.935	3.640	0.881	5.288	0.958	6.094	0.963	7.790	1.823	0.000	0.444	0.130	0.001	0.493	0.261
	* Lachnospiraceae*	*Coprococcus*	2.337	0.381	2.565	0.577	3.196	0.430	2.058	0.266	1.667	0.443	2.671	0.305	2.511	0.251	2.528	0.346	0.667	0.138	0.275	0.730	0.190	0.405
		Uncl. *Lachnospiraceae*	3.529	1.549	3.199	1.183	2.996	0.520	2.694	0.384	2.478	0.401	2.073	0.362	3.370	0.427	2.877	0.403	0.392	1.000	0.521	0.503	1.000	0.650
		*Roseburia*	0.022	0.016	0.000	0.000	0.154	0.048	0.230	0.081	0.087	0.048	0.005	0.002	0.254	0.107	0.167	0.088	0.563	0.001	0.396	0.639	0.003	0.535
	Uncl. *Clostridiales*	Uncl. *Clostridiales*	2.626	0.400	2.474	0.540	4.814	0.865	2.431	1.201	2.495	0.504	5.306	0.963	4.146	0.582	4.678	0.443	0.039	0.060	0.046	0.056	0.091	0.110
	* Peptococcaceae*	*Peptococcus*	0.375	0.106	0.947	0.447	0.365	0.058	0.136	0.023	0.610	0.144	1.124	0.336	0.802	0.140	1.016	0.183	0.001	0.040	0.307	0.002	0.067	0.439
	* Peptostreptococcaceae*	Uncl. *Peptostreptococcaceae*	17.905	3.213	27.179	2.932	14.866	1.648	8.101	1.152	16.008	2.719	33.242	3.810	29.994	4.350	23.425	3.924	0.000	0.000	0.014	0.000	0.000	0.039
		*Peptostreptococcus*	0.103	0.085	0.010	0.003	0.006	0.003	0.005	0.002	0.962	0.643	0.811	0.388	0.025	0.010	0.025	0.009	0.006	0.040	0.107	0.011	0.067	0.232
		*Sporacetigenium*	0.024	0.010	0.057	0.019	0.573	0.149	0.271	0.167	0.023	0.008	0.307	0.189	0.389	0.181	0.176	0.144	1.000	0.004	0.482	1.000	0.008	0.618
	* Ruminococcaceae*	*Acetanaerobacterium*	0.139	0.047	0.161	0.064	0.326	0.038	0.091	0.013	0.540	0.288	0.488	0.149	0.507	0.163	0.738	0.083	0.000	0.796	0.391	0.000	0.829	0.535
		*Butyricicoccus*	0.088	0.032	0.025	0.011	0.164	0.037	0.121	0.023	0.287	0.086	0.203	0.066	0.144	0.023	0.158	0.028	0.000	0.332	0.039	0.000	0.386	0.097
		*Faecalibacterium*	0.138	0.057	0.219	0.107	0.673	0.148	2.224	0.251	0.298	0.135	0.372	0.129	0.743	0.079	0.822	0.102	0.018	0.000	0.000	0.029	0.000	0.000
		*Oscillibacter*	0.033	0.024	0.004	0.002	0.351	0.071	0.112	0.030	0.050	0.023	0.016	0.009	0.152	0.041	0.103	0.027	0.064	0.000	0.003	0.088	0.000	0.010
		Uncl. *Ruminococcaceae*	0.638	0.289	0.192	0.101	2.204	0.433	0.505	0.096	2.817	0.937	1.696	0.431	3.020	0.358	2.568	0.253	0.000	0.006	0.425	0.000	0.012	0.559
		*Ruminococcus*	0.000	0.000	0.006	0.006	1.227	0.237	0.150	0.080	0.003	0.002	0.000	0.000	0.004	0.004	0.002	0.002	0.000	0.000	0.000	0.000	0.000	0.000
		*Subdoligranulum*	0.022	0.018	0.106	0.032	0.782	0.307	0.482	0.125	0.007	0.006	0.001	0.001	0.002	0.001	0.002	0.002	0.000	0.002	0.002	0.000	0.005	0.008
	* Veillonellaceae*	*Acidaminococcus*	1.188	0.733	0.330	0.263	0.063	0.044	0.234	0.126	0.004	0.002	0.001	0.001	0.001	0.001	0.001	0.001	0.065	0.255	0.205	0.088	0.304	0.342
		*Allisonella*	0.202	0.051	0.054	0.019	0.064	0.011	0.071	0.017	0.174	0.037	0.374	0.092	0.039	0.007	0.084	0.018	0.013	0.000	0.000	0.022	0.000	0.000
		*Megamonas*	1.672	0.664	2.058	1.760	0.685	0.306	1.933	0.624	3.517	1.346	1.712	0.710	1.031	0.331	0.608	0.113	0.521	0.182	0.273	0.629	0.233	0.405
		*Megasphaera*	10.199	5.284	10.947	4.906	11.910	2.905	14.285	3.222	0.480	0.234	0.014	0.009	0.001	0.000	0.167	0.150	0.000	0.875	1.000	0.000	0.893	1.000
		Uncl. *Veillonellaceae*	0.104	0.024	0.020	0.009	0.199	0.030	0.186	0.019	0.181	0.034	0.059	0.022	0.107	0.030	0.102	0.021	1.000	0.000	0.000	1.000	0.000	0.000
		*Phascolarctobacterium*	1.968	0.470	0.164	0.103	4.067	0.421	6.611	0.479	4.284	0.553	1.442	0.641	3.242	0.217	4.547	0.861	0.863	0.000	0.000	0.899	0.000	0.000
	* Erysipelotrichaceae*	Uncl. *Erysipelotrichaceae*	1.428	0.372	2.259	0.418	1.375	0.204	0.564	0.147	1.196	0.387	1.195	0.332	0.260	0.073	0.198	0.085	0.001	0.000	0.261	0.001	0.000	0.405
		*Turicibacter*	0.001	0.001	0.042	0.042	0.552	0.398	0.128	0.104	0.000	0.000	0.000	0.000	0.015	0.010	0.005	0.004	0.039	0.105	0.189	0.056	0.154	0.338
***Fusobacteria***																								
	* Fusobacteriaceae*	*Cetobacterium*	0.002	0.002	0.000	0.000	0.007	0.004	0.009	0.008	0.728	0.717	1.264	1.024	0.011	0.006	0.006	0.006	0.009	0.228	0.559	0.015	0.278	0.650
		*Fusobacterium*	0.857	0.459	0.126	0.063	0.262	0.169	0.194	0.080	11.495	2.651	8.440	1.950	3.282	0.941	3.716	1.438	0.000	0.002	0.005	0.000	0.005	0.016
		Uncl. *Fusobacteriaceae*	0.985	0.829	0.049	0.041	0.869	0.413	0.562	0.190	4.854	1.419	1.175	0.363	1.671	0.471	2.769	0.798	0.000	0.010	0.204	0.000	0.019	0.342
***Proteobacteria***																								
	* Alcaligenaceae*	*Sutterella*	0.236	0.089	0.020	0.017	0.527	0.074	0.547	0.056	0.957	0.359	0.066	0.020	0.465	0.184	0.222	0.080	0.306	0.000	0.001	0.402	0.000	0.003
	* Desulfovibrionaceae*	*Desulfovibrio*	0.129	0.041	0.043	0.025	0.263	0.055	0.110	0.029	0.533	0.159	0.279	0.107	0.248	0.108	0.212	0.121	0.004	0.141	0.167	0.008	0.190	0.320
	* Campylobacteriaceae*	*Campylobacter*	0.637	0.587	0.007	0.005	0.015	0.007	0.013	0.005	0.021	0.011	0.004	0.002	0.010	0.006	0.003	0.002	0.686	0.576	0.868	0.730	0.613	0.904
	* Succinivibrionaceae*	*Anaerobiospirillum*	0.752	0.470	0.017	0.005	0.178	0.051	0.099	0.037	0.835	0.259	0.298	0.131	0.531	0.206	0.075	0.020	0.686	0.002	0.868	0.730	0.005	0.904
		*Succinivibrio*	2.147	1.127	1.545	0.991	8.204	1.911	2.035	0.335	0.732	0.253	0.025	0.007	1.857	0.683	0.953	0.465	0.000	0.000	0.011	0.000	0.000	0.033
	* Enterobacteriaceae*	*Escherichia/Shigella*	0.555	0.476	0.140	0.090	0.032	0.025	0.123	0.059	0.670	0.271	1.651	1.153	0.026	0.015	0.180	0.131	0.541	0.459	0.638	0.629	0.499	0.708
***Spirochaetes***																								
	* Spirochaetaceae*	*Treponema*	0.000	0.000	0.000	0.000	0.570	0.273	0.030	0.015	0.000	0.000	0.000	0.000	0.061	0.035	0.020	0.013	0.018	0.000	0.000	0.029	0.000	0.000
**UNCLASSIFIED**																								
		Uncl. *Bacteria*	0.096	0.032	0.044	0.006	0.084	0.023	0.036	0.009	0.132	0.020	0.116	0.027	0.136	0.046	0.102	0.028	0.009	0.219	1.000	0.015	0.274	1.000

*Results are presented as mean and associated standard error of the mean (SEM). Uncl. unclassified; FDR, false discovery rate*.

Unclassified *Peptostreptococcaceae* was a prominent taxonomic group across all ages and diets, ranging from a mean of 8.1% of the community in cats fed kibbled diets at 260 weeks, to 33.2% in cats fed the canned diet at 17 weeks.

In young cats (8 and 17 weeks) fed kibbled diets, unclassified *Peptostreptococcaceae* were the most relatively abundant taxa averaging 22.5% (Table 4). However, in older cats (104 and 260 weeks) on the same diet, *Prevotella* were the most dominant group, with an average relative abundance of 29.7%. While *Lactobacillus* was present in moderate abundance in the feces of young cats fed kibbled diets, abundance declined markedly (0.02%) in older cats fed kibbled diets.

In cats fed canned diets, unclassified *Peptostreptococcaceae* were also prominent at all ages, increasing from 16% at 8 weeks to approximately 30% at 17 and 104 weeks and decreasing to 23.4% at 260 weeks of age (Table 4). Other major taxa in cats fed canned diets included *Prevotella* (6.8–16%) and a group of unclassified *Prevotellaceae* (7.9–10.3%). *Clostridium* were also prominent in younger cats fed the canned diet (4.2 and 10.0% in 8 and 17 week old cats, respectively), although their abundances declined with age (1.9 and 3.2% in 104 and 260 weeks).

Overall, the unclassified *Peptostreptococcaceae* had greater relative abundances in cats fed the canned diet compared to the kibbled diet (averaging 17.0 and 25.7%, in the kibbled and canned diet, respectively; FDR < 0.05). *Prevotella* were more abundant in cats fed the kibbled diet, averaging 20.0% relative abundance, compared to 10.8% in the cats fed the canned diets. Similarly, *Megasphaera* (averaging 11.8 vs. 0.2% in the kibbled and canned diet, respectively; FDR < 0.05) and *Blautia* (averaging 9.6 vs. 5.7% in the kibbled and canned diet, respectively; FDR < 0.05) were more prevalent in cats fed the kibbled than the canned diet, respectively. Unclassified *Prevotellaceae* (averaging 1.2 vs. 8.1% in cats fed kibbled and canned diets, respectively; FDR < 0.05) and *Bacteroides* (1.8 vs. 7.3% in cats fed kibbled and canned diets, respectively; FDR < 0.05) were higher in cats fed canned diets.

### Dataset integration

**Figure 3:**
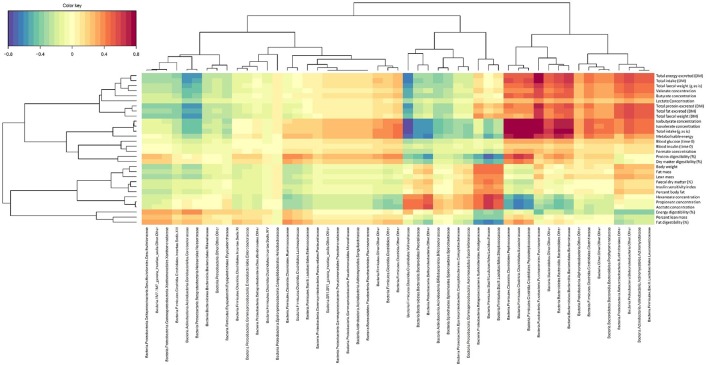
Canonical correlation heat map showing the relationships between fecal family-level bacterial taxa and physiological markers in domestic cats (*Felis catus*) fed the kibbled and canned diets at 260 weeks of age. Association measures were defined as: >|0.8| high, >|0.6| good, and >|0.5| moderate associations.

**Figure 4:**
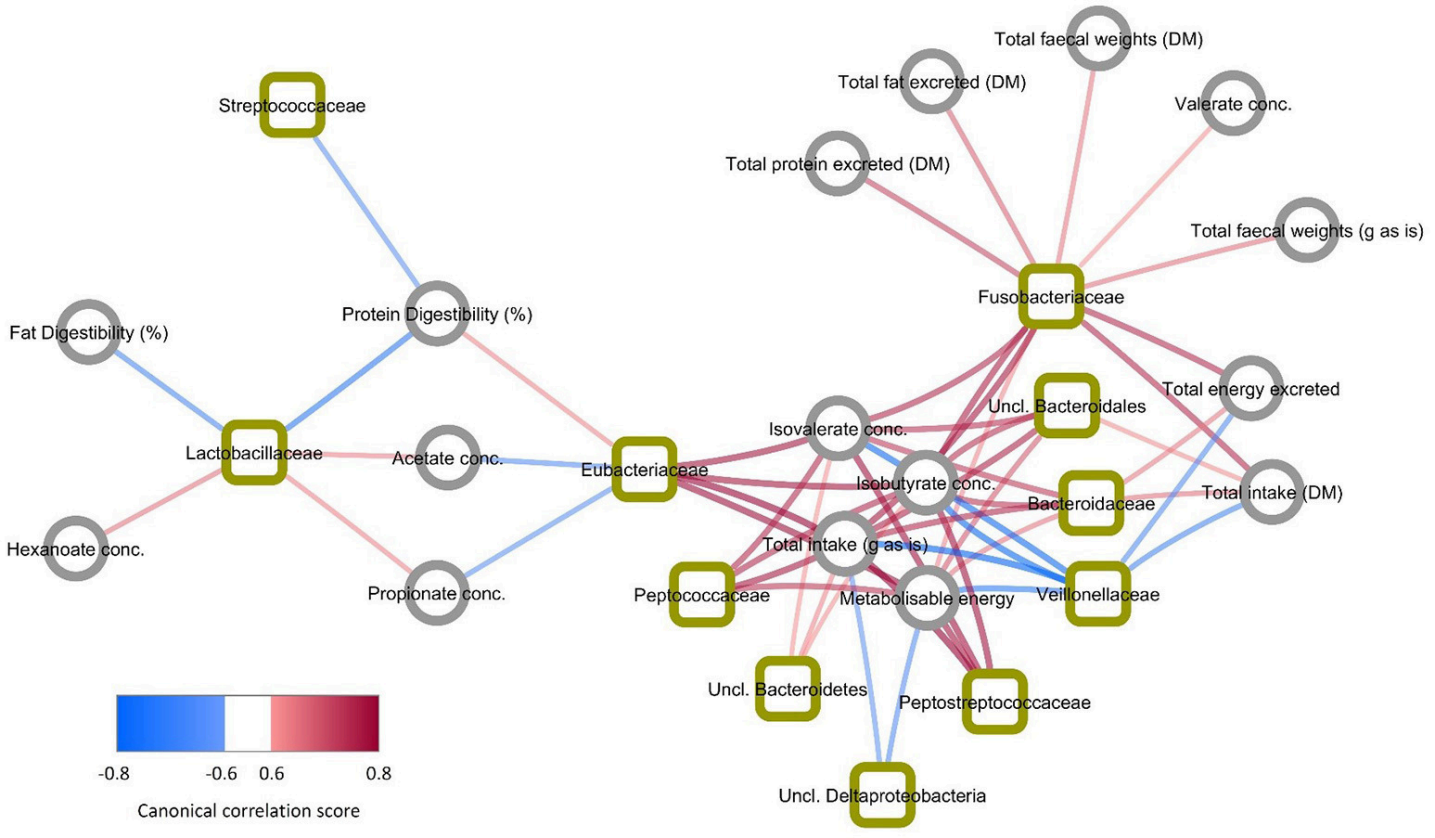
Network plot showing bacteria and physiological markers with the highest association measures (>|0.6|). Bacteria are indicated by squares and physiological markers and metabolites indicated by circles. Line colors indicate direction of association with blue showing negative association and red showing positive association. Line widths and shading are proportional to association measures, with increasing width and darker shading indicating higher absolute association.

The key relationships between microbial composition and physiological parameters in five year old (week 260) cats are shown in a Clustered Image Map (Figure 3) and a network plot (association measure cut-off 0.60 on 2 dimensions; Figure 4). Dataset integration is provided in Supplementary Material, Data Sheet 2.

Unclassified members of *Bacteroidetes, Bacteroidales*, and *Bacteroidaceae* were positively associated with isovalerate and isobutyrate concentrations (>0.64), total intake (>0.76), fecal gross energy concentrations (>0.58), and the Metabolizable energy (ME) content of the diet (>0.58). Unclassified *Bacteroidetes* and unclassified *Bacteroidales* were also positively associated with the excretion of crude protein, crude fat and gross energy (>0.52) and with intake (>0.70). Unclassified *Bacteroidetes* and unclassified *Bacteroidales* showed weak negative associations with acetate, propionate and hexanoate concentrations (< −0.48).

Members of *Eubacteriaceae* and *Peptostreptococcaceae* were positively associated with isovalerate and isobutyrate concentrations (>0.76) and negatively associated with acetate, and propionate (< −0.64). Both were also positively associated with the ME content of the diet (>0.76) and crude protein digestibility (>0.64 and >0.58 respectively). *Fusobacteriaceae* was also positively associated with isovalerate and isobutyrate, the excretion of crude protein, crude fat and gross energy and total fecal weight (>0.76). This family was also positively associated with intake (>0.76).

In contrast *Veillonellaceae* were negatively associated with isovalerate and isobutyrate concentrations and total intake (< −0.76) and fecal gross energy content (< −0.70). *Lactobacillaceae* were positively associated with acetate, propionate and hexanoate (>0.58) and negatively associated with the apparent digestibility of crude fat and crude protein (< −0.70). *Streptococcaceae* was negatively associated with crude protein and crude fat apparent digestibility (< −0.64). Unclassified *Deltaproteobacteria* were also positively associated with acetate (>0.58), propionate and hexanoate (>0.52) (Figure 3), and negatively associated with intake (< −0.58) and ME content (< −0.64) (Figure 4).

Glucose T0, insulin sensitivity index and body composition were poorly associated (< 0.40–0.46) with individual microbial taxa (Figure 3).

## Discussion

While previous studies have also shown that age affects the composition of microbiota in the domestic cat (Deusch et al., 2015; Masuoka et al., 2017), this is the first study to investigate the effects of both aging and diet on fecal microbiota in the same cohort of domestic cats over an extended (5 year) timeframe. We showed that both age and diet affected fecal microbiota, and while body composition and glycaemic response parameters varied with age, they did not vary significantly based on diet.

### Microbiome composition variation with age

We hypothesized that microbiota composition would be affected by age; indeed we saw over half of the observed taxa altering between kitten (8 and 17 weeks of age) and adult (5 years of age). A recent study investigated the fecal microbiome of cats fed a kibbled diet as they grew from 18 to 42 weeks of age (Deusch et al., 2015). *Lactobacillus* and *Bifidobacterium* are generally recognized as being beneficial to the host, and dominated when the kittens were younger (18 weeks), while *Bacteroides, Prevotella*, and *Megasphaera* were the dominant genera at 42 weeks of age. These results differ from culture-based studies where the researchers did not detect bifidobacteria in young kittens and showed lactobacilli abundance was similar in cats at various ages (Masuoka et al., 2017). They are, however, in agreement with our results showing a decreased relative abundance of *Lactobacillus* and *Bifidobacterium* and increased *Prevotella* and *Megasphaera* in aging cats fed kibbled diets. Interestingly, while *Lactobacillus* and *Bifidobacterium* were detected at a low frequency (0.001–0.002%) in kittens (8–17 weeks) fed the canned diets, they were not detected from these same cats at 5 years of age. The higher abundances of *Lactobacillus* and *Bifidobacterium* in the earlier times points (8–17 weeks) is likely due to the influence of milk-feeding/weaning schedule. The contrasting results between 16S rRNA gene based analyses in the current study, shotgun metagenome sequencing data (Deusch et al., 2015) and the culture-based techniques (Masuoka et al., 2017) will be at least in part due to differences in methodologies used. Given that the majority of taxa in complex microbiomes have not yet been cultured, culture-based analyses of microbiomes, which are often of relatively low depth, will be inherently biased. Sequencing strategies (e.g., 16S rRNA gene sequencing vs. metagenome sequencing) and platforms (e.g., 454 vs. Illumina) also introduce biases, but as we move away from sequencing partial 16S rRNA gene regions to shotgun sequencing approaches, it will be possible to more accurately determine both microbiome composition, and its functional potential, which will significantly advance our understanding of the pet microbiome.

Other research has suggested that enterococci, rather than the traditional *Bifidobacterium* and *Lactobacillus* species, may be important components for intestinal health in felines, due to their roles in lactic acid production (Masuoka et al., 2017). Both *Bifidobacterium* and *Lactobacillus* were detected in the current study; both were present at lower levels in the cats fed the canned diet and reduced with age regardless of diet. It has been reported that an increase in *Enterococcus* occurred in cats fed kibbled diets between 18 and 42 weeks of age, whereas the current study shows a reduction in the relative abundance of *Enterococcus* as the cats age (Deusch et al., 2015). In cats and dogs *Enterococcus* spp. have been linked with antimicrobial resistance (Iseppi et al., 2015; Abdel-Moein et al., 2017), however specific strains of *Enterococcus faecium* do appear to have probiotic potential in the cat (Bybee et al., 2011).

In addition to *Bifidobacterium* and *Lactobacillus, Faecalibacterium*, specifically *Faecalibacterium prausnitzii*, have been shown to be important for the health of the intestine (Rajilić-Stojanović and de Vos, 2014). *Faecalibacterium* have been reported in healthy cats fed a range of diets (Hooda et al., 2013; Suchodolski et al., 2015; Young et al., 2016). Hooda et al. (2013) observed no age effect on the relative abundance of *Faecalibacterium* when comparing 8, 12, and 16 week old kittens. Our results indicate an increased abundance of *Faecalibacterium* in 5 year old cats whereas another study showed a reduction in *Faecalibacterium* spp. in cats >10 years of age (Bell et al., 2014). Recent results have shown that *Faecalibacterium* abundance tends to be greater in obese (vs. lean) cats (Fischer et al., 2017).

### Microbiome composition varies with diet

We hypothesized that dietary format would impact microbial composition. We observed large changes in microbial composition in relation to diet. To better understand the changes in microbial composition we integrated them with a number of parameters including diet composition and related physiology parameters such as macronutrient digestibility. This analysis identified nine bacterial families that were associated with parameters relating to gross energy, crude protein and crude fat levels in the diet and their apparent digestibility, fecal organic acid concentrations and fecal DM. These families are mainly members of the phyla *Bacteroidetes* (*Bacteroidaceae*), *Firmicutes* (*Peptostreptococcaceae, Eubacteriaceae, Peptococcaceae*), and *Fusobacteria* (*Fusobacteriaceae*).

In our study, the only genus represented among the *Bacteroidaceae* was *Bacteroides*, which were relatively more abundant in the cats fed the canned diets, in agreement with a previous study from our laboratory (Bermingham et al., 2013b). Members of the *Bacteroidaceae* including *Bacteroides* have been observed in the feces of healthy cats (Deusch et al., 2015; Suchodolski et al., 2015; Fischer et al., 2017). While *Bacteroides* spp. can utilize a range of nitrogen and carbon sources (Hanning and Diaz-Sanchez, 2015) *Bacteroides* were associated with carbohydrate digestion in the dog (Bermingham et al., 2017). This contrasts the results observed in the current study, whereby positive associations between *Bacteroidaceae* and markers of crude protein digestion (protein digestibility, protein content of feces, fecal isobutyrate, and isovalerate) were observed. While this suggests that in the domestic cat, *Bacteroidaceae* are associated with crude protein digestibility, studies that have investigated either high protein diets (Hooda et al., 2013), or whole prey diets did not report this family (Kerr et al., 2014).

A group consisting of unclassified *Peptostreptococcaceae* were the dominant taxa observed in this study, irrespective of age or diet. This is similar to previous studies (Bermingham et al., 2013b; Fischer et al., 2017), but contrasts with other studies in healthy cats who show either low levels (Bell et al., 2014; Kerr et al., 2014; Suchodolski et al., 2015), or do not report this taxonomic group (Hooda et al., 2013; Deusch et al., 2015). While the role of *Peptostreptococcaceae* is the gut is not clear, their presence has been associated with a range of illnesses in humans, including non-alcoholic fatty liver disease (Jiang et al., 2015) and Crohn's disease (Pascal et al., 2017). However, because of their prominence in the fecal community of the cats in our study, all of which remained healthy, it would seem unlikely that they have a deleterious effect in cats. In the dog, *Peptostreptococcaceae* are associated with fecal protein content; in the current study they were associated with metabolites of protein fermentation, namely isovalerate, and isobutyrate. This suggests that *Peptostreptococcaceae* may perform a similar role in cats and dogs.

*Eubacteriaceae* was mainly represented by members of *Eubacterium* in the current study. *Eubacterium* spp. have been observed in healthy cats of various life stages (Bermingham et al., 2013b; Hooda et al., 2013; Bell et al., 2014; Kerr et al., 2014; Deusch et al., 2015; Suchodolski et al., 2015; Fischer et al., 2017; Masuoka et al., 2017). While *Eubacterium* is phenotypically heterogeneous, and known to utilize both carbohydrate and protein sources, in the human gut, it is generally regarded as a carbohydrate fermenter, producing acetate, formate, succinate, and lactate (Hanning and Diaz-Sanchez, 2015). Some *Eubacterium* members are also known to be prominent butyrate producers in the human gut (Louis and Flint, 2009). However, in the current study *Eubacteriaceae* were positively associated with crude protein digestibility and fermentative products from protein breakdown (isovalerate and isobutyrate) and negatively associated with markers of carbohydrate fermentation (acetate, propionate and butyrate) suggesting a role in protein fermentation in the cat. This may explain the higher abundance of in *Eubacterium* observed in cats fed high protein diets (Hooda et al., 2013). Interestingly, in the dog, *Eubacteriaceae* was associated with carbohydrate and dry matter content of the diets (Bermingham et al., 2017).

Members of *Peptococcaceae*, including *Peptococcus* have been observed in the feces of healthy cats (Bermingham et al., 2013b; Bell et al., 2014; Suchodolski et al., 2015) and show a tendency to decrease in abundance in cats with diabetes (Bell et al., 2014) and diarrhea (Suchodolski et al., 2015). In the current study, *Peptococcaceae* (*Peptococcus*), as with *Eubacteriaceae* and *Peptostreptococcaeae*, were positively associated with crude protein digestibility and markers of protein fermentation. In the dog *Peptococcaceae* was also associated with crude protein content of the diet and crude protein digestibility (Bermingham et al., 2017).

In the dog, *Fusobacteriaceae* have been shown to be negatively associated with fecal crude protein content (Bermingham et al., 2017), however, in this study unclassified *Fusobacteriaceae* and *Fusobacterium* were positively associated with food intake, gross energy and crude protein content of the feces and the fecal concentrations of isobutyrate and isovalerate. Members of the *Fusobacteria* phylum have been previously observed in healthy cats (Bermingham et al., 2013b; Bell et al., 2014; Suchodolski et al., 2015; Fischer et al., 2017) and their abundance is higher when fed high protein diets (Hooda et al., 2013). In humans, *Fusobacterium* spp. can utilize amino acids to produce butyrate (Hanning and Diaz-Sanchez, 2015); in the current study we observed a strong association between *Fusobacteriaceae* and butyrate, suggesting that in this could be an important contributor of intestinal butyrate production in the cat.

The cat differs from the dog in that it is an obligate carnivore rather than a facultative carnivore. In the dog we have observed that *Clostridiaceae* were positively associated with crude protein digestibility (Bermingham et al., 2017). In the current cat study *Eubacteriaceae* and *Peptostreptococcaceae* showed strong associations with crude protein digestibility, whereas *Clostridiaceae* were only weakly associated. Similarly, *Ruminococcaceae* abundance in the dog were positively associated with short-chain fatty acids (Bermingham et al., 2017), whereas in the cat they were negatively associated. The differences between the cat and dog in how their microbiota respond to different feed ingredients is of considerable interest.

### Impact of diet on body fat composition

We hypothesized that when cats were *ad libitum* fed, dietary format would affect body composition. Surprisingly, our cats had similar body composition regardless of dietary treatment and had a normal body condition score. As recently reviewed, the prevalence of obesity is increasing with age (5–10 years of age), and identified that neuter status (neutered) was a major risk factor in developing obesity (Tarkosova et al., 2016). The impact of diet on obesity is more complicated with some studies showing increased risks associated with kibble diets (Scarlett et al., 1994; Rowe et al., 2015) and others with canned diets (Russell et al., 2000). A recent study comparing lean and obese neutered cats on the same diet showed that in neutered cats, obesity led to increased abundances of *Bulleida, Prevotella, Acidaminococcus, Faecalibacterium* and *Phascolarctobacterium* and decreased abundances of *Blautia* and *Clostridium* (Fischer et al., 2017). In the current study we observed weak positive associations between microbiota and fat mass and % body fat with *Alcaligenaceae* (*Sutterella*), *Lactobacillaceae* (*Lactobacillus*), and *Streptococcaceae* (*Lactococcus, Streptococcus*). However, we did observe greater abundance *Prevotella, Acidaminococcus* and *Faecalibacterium* and decreased *Blautia* and *Clostridium* in cats fed the kibbled diet, suggesting a similar pattern to obese cats.

Obesity arises as a mismatch between energy intake and energy expenditure. Certainly, indoor living (therefore reduced physical activity) has also been identified as a risk factor for obesity in domestic cats (Rowe et al., 2015; Öhlund et al., 2017). The cats in the current study were colony housed and therefore would have had reduced physical activity compared to free-ranging domestic cats. Interestingly, some studies have indicated that increased water content promotes physical activity (Deng et al., 2014). However, this finding was not replicated in our colony (Thomas et al., 2017). While obesity has been observed in young, growing cats (Häring et al., 2013), it may be possible that the cats in the current study may develop changes in body composition as they continue to age.

### Impact of diet on insulin sensitivity

When cats develop Type 2 diabetes, they become insulin resistant and therefore have lowered insulin sensitivity. We hypothesized that both age and diet would affect insulin sensitivity in the cat, however, we observed no effect of age or diet on insulin sensitivity index. A comparison of insulin sensitivity indices between cats fed kibbled and canned diets at the 260 week time point appeared to show a segregation of insulin sensitivity values between diets, with higher values in cats fed canned diets. However, differences in insulin sensitivity were not significant between post-weaning diet at this time point (permutation MANOVA *P* = 0.096). Nonetheless, significant correlations were observed between two related taxa, *Streptococcus* and *Lactobacillus*, and insulin sensitivity index (*R* > 0.59, *P* < 0.02), suggesting a possible link between some members of the intestinal community and insulin response. A recent study investigating the fecal microbiota of cats with diabetes and treated with insulin, showed no significant changes in fecal microbial composition although the abundances of *Bacterioides* and *Bifidobacterium* trended (*P* < 0.10) toward decreasing with the disease (Bell et al., 2014). Similarly, in humans there are few compositional differences in humans with Type 2 diabetes (Yassour et al., 2016), however the function of the microbiota is markedly different (Zhang et al., 2013).

Previous studies in the cat have indicated that identified that insulin sensitivity is correlated to body condition score (Häring et al., 2013). Furthermore, recent epidemiological evidence suggests that kibbled diets are a risk factor for diabetes in non-obese cats older than 10 years of age (Öhlund et al., 2017). Therefore, the lack of effect observed in the current study may be due to either the similar body condition score and/or the relatively young age of our cats, and it will be of interest to follow their progress as the cats mature further (>7 years old).

## Conclusions

This study showed that diet and age both affect fecal microbial composition, but neither appear to impact on body composition and markers of glycaemic response in adult aged (5 year old) cats. This study will continue to assess these parameters as the cats grow older. Microbial families including *Peptostreptococcaeae* and *Eubacteriaceae*, and to a lesser extent *Fusobacteriaceae* and *Peptococcaceae* appear to be key in parameters related to crude protein digestion in the cat.

## Author contributions

The experimental design was devised by EB and DT. Animal work was undertaken by EB, DT, CB, and NC. Laboratory work was undertaken by CB and DR. Bioinformatic and statistical analyses were conducted by EB, WY, and CM and dataset integration was undertaken by PM. All authors contributed to the preparation of the manuscript.

### Conflict of interest statement

The authors declare that the research was conducted in the absence of any commercial or financial relationships that could be construed as a potential conflict of interest.

## Supplementary material

Data Sheet 1 R codes for microbial analysis.

Data Sheet 2 R codes for dataset intergration.

## Abbreviations

AAFCO: Association of American Feed Control Officials
ANOVA: analysis of variance
AUC: area under the curve
CCA: canonical correlation analysis
DM: dry matter
FDR: false discovery rate
FID: flame ionization detector
GC: gas chromatography
IRMS: isotope-ratio mass spectrometry
IVGTT: intravenous glucose tolerance test
IVITT: intravenous insulin tolerance test
MS: mass spectrometry
ME: Metabolizable energy
NFE: nitrogen free extractables
OTU: operational taxonomic units
PCA: principal component analysis
RDP: Ribosomal Database Project
REML, restricted (or residual: or reduced) maximum likelihood
SEM: standard error of the mean
TC: Thermal Chromatography.
