# Supplementary material for: The Fecal Microbiota in the Domestic Cat (Felis catus) Is Influenced by Interactions Between Age and Diet; A Five Year Longitudinal Study
Source: Front Microbiol. 2018 Jun 19;9:1231. doi: 10.3389/fmicb.2018.01231 (PMC6018416; doi:10.3389/fmicb.2018.01231)
Supplement: Data Sheet 1 — R codes for microbial analysis. [file Data_Sheet_1.DOCX]

###############################

###############################

###############################

####

#### Qiime 1.8 scripts

####

#### Identify chimeras and filter out

####

identify_chimeric_seqs.py -i seqs_joined.fna -m usearch61 -o usearch_checked_chimeras/ -r /dataset/qiime_taxonomies/active/gg_13_8/rep_set/97_otus.fasta

filter_fasta.py -f seqs_joined.fna -o seqs_chimeras_filtered.fna -s usearch_checked_chimeras/chimeras.txt -n

#### Pick otus

####

pick_otus.py -m usearch61 -i seqs_chimeras_filtered.fna -o usearch61_picked_otus/

#### Pick representative sequences from otus

####

pick_rep_set.py -i usearch61_picked_otus/seqs_chimeras_filtered_otus.txt -f seqs_chimeras_filtered.fna -o usearch61_picked_otus/rep_set.fna

#### Align sequences

####

align_seqs.py -i usearch61_picked_otus/rep_set.fna -t /dataset/qiime_taxonomies/active/gg_13_8/rep_set_aligned/97_otus.fasta -o usearch61_picked_otus/pynast_aligned_defaults

#### Assign taxonomies using rdp

####

assign_taxonomy.py -i usearch61_picked_otus/rep_set.fna -m rdp

filter_alignment.py -i usearch61_picked_otus/pynast_aligned_defaults/rep_set_aligned.fasta -m /dataset/qiime_taxonomies/active/gg_13_5/lanemask_in_1s_and_0s.txt -o usearch61_picked_otus/filtered_alignment/

#### Make phylogentic tree

####

make_phylogeny.py -i usearch61_picked_otus/filtered_alignment/rep_set_aligned_pfiltered.fasta -o usearch61_picked_otus/filtered_alignment/rep_phylo.tre

#### Create otu table

####

make_otu_table.py -i usearch61_picked_otus/seqs_chimeras_filtered_otus.txt -o usearch61_picked_otus/otu_table_non_chimeric.biom -t rdp_assigned_taxonomy/rep_set_tax_assignments.txt

biom summarize-table -i usearch61_picked_otus/otu_table_non_chimeric.biom -o library_stats.txt

#### Make taxonomy tables

####

summarize_taxa_through_plots.py -i usearch61_picked_otus/otu_table_non_chimeric.biom -m mapping.txt -o taxa_summary -s

#### Calculate beta diversity, e value 16052

####

beta_diversity_through_plots.py -i usearch61_picked_otus/otu_table_non_chimeric.biom -o bdiv_even16052/ -t usearch61_picked_otus/filtered_alignment/rep_phylo.tre -m mapping.txt -e 16052

#############################################

#############################################

#############################################

####

#### R scripts for statistical analyses

####

####

#### Read mapping file

####

mapping <- data.frame(read.delim(file="mapping.txt", sep="\t",stringsAsFactors=F))

mapping <- mapping[order(mapping[,1]),]

#### Read taxonomy table (from Qiime output)

####

L6 <- data.frame(read.delim(file="otu_table_non_chimeric_sorted_L6.txt", sep="\t"))

rownames(L6) <- L6[,1]

L6 <- subset(L6, select=-Taxon)

L6 <- L6[,order(colnames(L6))]

colnames(L6)

#### Permutation ANOVA

####

library(lmPerm)

grp1 <- "Post_Diet"

fact1 <- as.factor(mapping[,grp1])

grp2 <- "Week"

fact2 <- as.factor(mapping[,grp2])

grp3 <- "Name"

stats.table <- as.data.frame(matrix(1, ncol = 3, nrow = nrow(L6)))

colnames(stats.table) <- c(grp1,grp2,paste(grp1,"X",grp2,sep="_"))

i <- 1

for(i in c(1:nrow(L6))) {

reptest <- as.numeric(L6[i,])

stat.test <- unlist(summary(aovp(reptest ~ fact1 * fact2 + Error(grp3))))

stats.table[i,grp1] <- stat.test["Pr(Prob)1"]

stats.table[i,grp2] <- stat.test["Pr(Prob)2"]

stats.table[i,paste(grp1,"X",grp2,sep="_")] <- stat.test["Pr(Prob)3"] }

fdr.table <- stats.table

i <- 1

for(i in 1:ncol(fdr.table)) {

fdr.table[,i] <- p.adjust(fdr.table[,i], method="BH") }

########################################

########################################

########################################
