# Supplementary material for: The Fecal Microbiota in the Domestic Cat (Felis catus) Is Influenced by Interactions Between Age and Diet; A Five Year Longitudinal Study
Source: Front Microbiol. 2018 Jun 19;9:1231. doi: 10.3389/fmicb.2018.01231 (PMC6018416; doi:10.3389/fmicb.2018.01231)
Supplement: Data Sheet 2 — R codes for dataset intergration. [file Data_Sheet_2.DOCX]

## Install packages if they are not installed already:

install.packages("mixOmics")

install.packages("lmPerm")

install.packages("ggplot2")

install.packages("heatmaply")

install.packages("igraph")

## Load required packages:

library(mixOmics)

library(lmPerm)

library(ggplot2)

library(heatmaply)

library(igraph)

## Function to convert uncalculated correlations to zero:

na.zero <- function (x) {

x[is.na(x)] <- 0

return(x)

}

## Load in the data:

setwd($data)

Trt_info = read.table("Trt_info.txt",header=TRUE,sep="\t")

Phys_data = read.table("Phys_data.txt",header=TRUE,sep="\t")

Micro_data = read.table("Micro_data.txt",header=TRUE,sep="\t")

## Tidy up column names for physical data:

colnames(Phys_data)=c("Bodyweight","Lean Mass","Fat Mass","% fat","% lean","T0 Insulin µU/ml","T0 Glucose (mmol/l)","Ins Sens Index","Total intake (g as is)","Total faecal weights (g as is)","Total intake(DM)","Total faecal weights (DM)","DM Digestibility (%)","Faecal Dry Matter %","T energy excreted (DM)","Energy Digestibility (%)","T protein excreted (DM)","Protein Digestibility (%)","T Fat excreted (DM)","Fat Digestibility (%)","ME (kcal ME/kg food)","Formate","Acetate","Propionate","Isobutyrate","Butyrate","Isovalerate","Valerate","Hexanoate","Heptanoate","Lactate","Succinate")

# Data heatmaps - Strong separation on "post_diet" variables

## Phys data heatmap

Phys_data_a = read.table("Phys_data_averaged.txt",header=TRUE,sep="\t",row.names=1)

rownames(Phys_data_a)=c("Wet->Dry","Dry->Dry","Dry->Wet","Wet->Wet")

colnames(Phys_data_a)=c("Bodyweight","Lean Mass","Fat Mass","% fat","% lean","T0 Insulin µU/ml","T0 Glucose (mmol/l)","Ins Sens Index","Total intake (g as is)","Total faecal weights (g as is)","Total intake(DM)","Total faecal weights (DM)","DM Digestibility (%)","Faecal Dry Matter %","T energy excreted (DM)","Energy Digestibility (%)","T protein excreted (DM)","Protein Digestibility (%)","T Fat excreted (DM)","Fat Digestibility (%)","ME (kcal ME/kg food)","Formate","Acetate","Propionate","Isobutyrate","Butyrate","Isovalerate","Valerate","Hexanoate","Heptanoate","Lactate","Succinate")

heatmap.2(t(log2(as.matrix(Phys_data_a))),margins = c(10,10),tracecol = NULL,density.info="none")

## Micro_data data heatmap

Micro_data_a = read.table("Micro_data_averaged.txt",header=TRUE,sep="\t",row.names=1)

rownames(Micro_data_a)=c("Wet->Dry","Dry->Dry","Dry->Wet","Wet->Wet")

heatmap.2(t((as.matrix(Micro_data_a[,-nearZeroVar(Micro_data)$Position]))^(1/3)),margins = c(10,10),tracecol = NULL,density.info="none")

# PCA

## Phys data PCA scree plot

plot(pca(Phys_data[,-nearZeroVar(Phys_data)$Position],ncomp=10),scree.type = "barplot")

## Phys data PCA variate plot - As shown in the scree plot, the first component explains most of the data with clear separation of the "post-diet" variable with virtually no separation on the "pre-diet", except in the "pre-diet" conditions assicated with the wet diet. Colours: Blue, Dry diet then Dry diet; Grey: Wet diet then Dry diet; Green: Wet diet then Wet diet; Orange: Dry diet then Wet diet.

plotIndiv(pca(Phys_data[,-nearZeroVar(Phys_data)$Position]),ind.names=Trt_info$Cat,group=as.numeric(as.factor(paste(Trt_info$Pre_diet,Trt_info$Post_Diet))),cex=5)

## Micro data PCA scree plot - The first component explains much more of the data than the others. We keep the second component for plotting purposes

plot(pca(Micro_data[,-nearZeroVar(Micro_data)$Position],ncomp=10),scree.type = "barplot")

## Micro data Micro_data (species) PCA - The microbiome data did not separate as well as the Physical data, but the "post-diet" separation seen in the physical data remains. Colours: Blue, Dry diet then Dry diet; Grey: Wet diet then Dry diet; Green: Wet diet then Wet diet; Orange: Dry diet then Wet diet.

plotIndiv(pca(Micro_data[,-nearZeroVar(Micro_data)$Position]),,ind.names=Trt_info$Cat,group=as.numeric(as.factor(paste(Trt_info$Pre_diet,Trt_info$Post_Diet))),cex=5)

# Correlations

## CIM -Before applying canonical component analysis, we check the correlations.

cim(na.zero(cor(Phys_data[c(-7,-15),-nearZeroVar(Phys_data[c(-7,-15),])$Position],Micro_data[c(-7,-15),-nearZeroVar(Micro_data[c(-7,-15),])$Position],use="complete.obs")),mar=c(34,12))

## Interactive heatmap

heatmaply(round(na.zero(cor(Phys_data[c(-7,-15),-nearZeroVar(Phys_data[c(-7,-15),])$Position],Micro_data[c(-7,-15),-nearZeroVar(Micro_data[c(-7,-15),])$Position],use="complete.obs")),2),mar=c(80,80,50,50))

# Shrunk RCC

rcc.shrink=rcc(Phys_data[-c(7,15),-c(30,32)],Micro_data[-c(7,15),-nearZeroVar(Micro_data[-c(7,15),])$Position],ncomp = 3, method ="shrinkage")

## Scree plot

plot(rcc.shrink, scree.type = "barplot")

## Variate plots - There is again, separation of the "post-diet" variable with virtually no separation on the "pre-diet", except in the "pre-diet" conditions assicated with the wet diet. Colours: Blue, Dry diet then Dry diet; Orange: Wet diet then Dry diet; Green: Wet diet then Wet diet; Grey: Dry diet then Wet diet.

plotIndiv(rcc.shrink,group=paste(Trt_info$Post_Diet[-c(7,15)],Trt_info$Pre_diet[-c(7,15)]),ind.names=Trt_info$Cat[-c(7,15)],cex=5)

## Circle plots

plotVar(rcc.shrink, comp = 1:2, cutoff = 0.7, var.names = c(TRUE, TRUE), cex = c(5, 5))

## CIM plot

rcc.shrink.association.measures =cim(rcc.shrink,margins=c(34,12))

## Interactive heatmap of association measures

heatmaply(round(as.matrix(rcc.shrink.association.measures$mat),2),mar=c(80,80,50,50))

## Write association measures to a file

write.table(rcc.shrink.association.measures$mat,"association_measures.txt",sep="\t")

## Network plot

rcc.shrink.net.result.0.6 = network(rcc.shrink,cutoff=0.6,interactive = FALSE)

write.graph(rcc.shrink.net.result.0.6$gR, file = "rcc.shrink.net.result.0.6.gml", format = "gml")
